# European Invasion of North American *Pinus strobus* at Large and Fine Scales: High Genetic Diversity and Fine-Scale Genetic Clustering over Time in the Adventive Range

**DOI:** 10.1371/journal.pone.0068514

**Published:** 2013-07-10

**Authors:** Bohumil Mandák, Věroslava Hadincová, Václav Mahelka, Radka Wildová

**Affiliations:** 1 Institute of Botany, Academy of Sciences of the Czech Republic, Průhonice, Czech Republic; 2 Faculty of Environmental Sciences, Czech University of Life Sciences Prague, Prague, Czech Republic; 3 Department of Ecology and Evolutionary Biology, University of Michigan, Ann Arbor, Michigan, United States of America; CNRS/Université Joseph-Fourier, France

## Abstract

**Background:**

North American *Pinus strobus* is a highly invasive tree species in Central Europe. Using ten polymorphic microsatellite loci we compared various aspects of the large-scale genetic diversity of individuals from 30 sites in the native distribution range with those from 30 sites in the European adventive distribution range. To investigate the ascertained pattern of genetic diversity of this intercontinental comparison further, we surveyed fine-scale genetic diversity patterns and changes over time within four highly invasive populations in the adventive range.

**Results:**

Our data show that at the large scale the genetic diversity found within the relatively small adventive range in Central Europe, surprisingly, equals the diversity found within the sampled area in the native range, which is about thirty times larger. Bayesian assignment grouped individuals into two genetic clusters separating North American native populations from the European, non-native populations, without any strong genetic structure shown over either range. In the case of the fine scale, our comparison of genetic diversity parameters among the localities and age classes yielded no evidence of genetic diversity increase over time. We found that SGS differed across age classes within the populations under study. Old trees in general completely lacked any SGS, which increased over time and reached its maximum in the sapling stage.

**Conclusions:**

Based on (1) the absence of difference in genetic diversity between the native and adventive ranges, together with the lack of structure in the native range, and (2) the lack of any evidence of any temporal increase in genetic diversity at four highly invasive populations in the adventive range, we conclude that population amalgamation probably first happened in the native range, prior to introduction. In such case, there would have been no need for multiple introductions from previously isolated populations, but only several introductions from genetically diverse populations.

## Introduction

Many studies of plant invasions have focused on comparative aspects of ecology, with emphases on understanding either the properties of species that determine their invasive potential [1]–[3] or the properties of communities that determine their resistance to invasion [4], [5]. The outcome of an invasion may be influenced by abiotic factors such as resource availability [6] or type and frequency of disturbance [7], and by biotic characteristics such as propagule supply [8], [9] or the abundance of natural enemies, competitors or mutualists in the host community [10], [11]. Although some general theories of invasibility have been put forward [6], [12], [13], it is not easy to come up with general explanations as to why some invasions succeed while other fail [3], [10], [14], [15]. This is because of the numerous factors influencing populations, species, plant communities, and whole ecosystems [14], [15].

Even if introduced species encounter physical environmental (e.g. climatic or edaphic) conditions in their adventive ranges that are similar to those in their native ranges, they face new biotic environments [14]. Therefore, the success of invasive species may largely depend on their ability to evolve in response to new environments, although adaptation may not always be a prerequisite for successful establishment of introduced populations [16]–[18]. The potential for local adaptation would seem to be limited by loss of genetic diversity during the introduction process and subsequent range expansion in newly colonized areas [19]. In particular, during colonization the founder effect may reduce genetic diversity in newly established populations relative to the source population [20].

Several mechanisms by which invasive populations can retain sufficient genetic diversity to enable adaptation have been suggested. First, rapid population expansion can occur immediately after an introduction, allowing retention of genetic diversity [21] and facilitating later adaptation to a local environment. Second, polyploidization and hybridization phenomena may rapidly produce novel diversity in the introduced area [22]. Finally, when a species is introduced to a new range from several genetically different populations, further recombination within the adventive range may dramatically increase genetic diversity outside the native range [23]–[27]. This population admixture among genetically distinct lineages has been predicted to contribute to invasion success by directly increasing fitness through hybrid vigour or by enhancing evolutionary potential within populations [28].

The time taken for introduced populations to acquire sufficiently high frequencies of adaptive genotypes might explain the substantial time lags (of years or decades) between initial establishment and the manifestation of invasiveness. These time lags have been well documented [29]–[32] and could be due to these evolutionary processes as well as ecological dynamics such as the time needed to spread to favourable habitat or to reach population sizes capable of producing abundant offspring [33].

Although many studies have reported multiple introductions of invasive species, the impacts of admixture on the fitness of invasive species have rarely been studied. Those studies that have been done have all shown a strong influence of the resulting genetic diversity on colonization success [28], [34]–[37]. Population-level genetic diversity appears important for long-term stability, especially if adaptive genetic variation is maintained along with neutral variation [38]. Following population establishment, genetic diversity may increase with population age due to admixture of populations derived from different introduction sources. These processes have been studied using theoretical models [39], but the amount of empirical information we have about the relative importance of spread rate and population age for the distribution of genetic diversity within and among invasive populations is still limited. It is nevertheless important to understand the distribution of genetic diversity along invasion routes, especially if we are to better understand invasion dynamics. However, we are usually unable to observe the invasion process as it happens because we cannot study the originally introduced individuals which had started the invasion, often many years earlier. Their progeny, which can slowly adapt to the new environment, also disappear over time in most cases. There is one prominent exception, however: trees. Invasive tree species represent a unique opportunity to study, in ideal cases, representatives of all generations that took part in an invasion within an area. The originally introduced trees and their progeny represent various stages of the lag phase. Thanks to the long life history of trees, a continuous record can be available at study sites, so the entire invasion history can be readily determined by using selectively neutral markers.

It has been shown that not only genetic diversity *per se* but also its distribution in space is an important aspect of population dynamics ([40] and references therein). On the one hand, it has been shown that spatial genetic structure (hereafter SGS) within populations is influenced by patterns of seed dispersal [41], mating system, [42] mating pattern [43], colonization events [44]–[46], competition [47], [48], demographic structure [49] and microhabitat heterogeneity [50]. On the other hand, SGS may itself have an effect on population dynamics by affecting the level of inbreeding and microenvironmental adaptation [50], mating pattern [43], or patterns of viability selection [51], [52]. Therefore, in newly established invading populations, characterizing both the patterns of fine-scale genetic diversity and SGS and their changes over time provides an important tool especially for shedding light on the processes of invasion when genetic reorganization can be expected. Analyses of both genetic diversity and SGS changes over time in tree species are quite rare, however [40].

From this standpoint, *Pinus strobus* in the Czech Republic is one of the best examples of an invasive tree under study [53]. Large amounts of this species’ seeds intended for cultivation have been imported from North America since the end of the 18^th^ century [54]. Historical records provide evidence of about tens of kilograms of *P. strobus* seeds introduced to different parts of the Czech Republic at the turn of the 18^th^ and 19^th^ century [54]. At present, this long-lived species is highly invasive in several mainly sandstone areas of the country [55] (further denoted as invasive populations; sensu Richardson *et al.* [56]), but non-invasive in other parts (further denoted as naturalized populations [56]). It is now a component not only of planted mixed forests but also of other forests, as well as occurring in sparsely vegetated rocky sites. In Central Europe, many sandstone areas are protected for their unique environment, and large-scale regeneration of an alien tree species therefore poses a problem of serious conservation concern [53].

We used *P. strobus* populations in the Czech Republic (Central Europe) and North America to test theoretical predictions concerning genetic diversity of an invasive species in its native and adventive ranges, and evolution in its adventive range on large as well as fine scales. In studying the pattern of large-scale genetic diversity, we specifically asked (1) whether there are any differences in population genetic diversity and structure between the native and the adventive ranges and between invasive and naturalized populations within the adventive range; (2) whether there is any indication of bottleneck in introduced populations in the adventive range, and (3) whether populations of *P. strobus* are derived from independent introduction events and from which areas. In a subsequent study we investigated the pattern of fine-scale genetic diversity and SGS and their changes over time within four highly invasive populations in the species’ adventive range. Such a fine-scale study might help clarify the species’ population dynamics at different localities over the course of its invasion history. In particular we: (1) investigated the pattern of genetic diversity within and among different age classes and in different highly invasive populations; and (2) asked if there is any SGS that is maintained within and among different age classes in different invasive populations.

## Materials and Methods

### Ethics Statement

The study did not involve endangered or protected species. To perform our study in the Bohemian Switzerland National Park, a collaboration agreement was made between the Institute of Botany of the Academy of Sciences of the Czech Republic and the National Park authorities. Other forests on the Czech side are managed by the Forests of the Czech Republic, State Enterprise, i.e. forests managed under public ownership, for which no special permission is required to collect small samples of needles from an alien invasive species. We did not procure special permissions for collecting in any of the US localities, as we collected just several needles from a very common tree species. We could confirm that none of the collection sites were privately owned.

### Study Species

**Figure 1:**
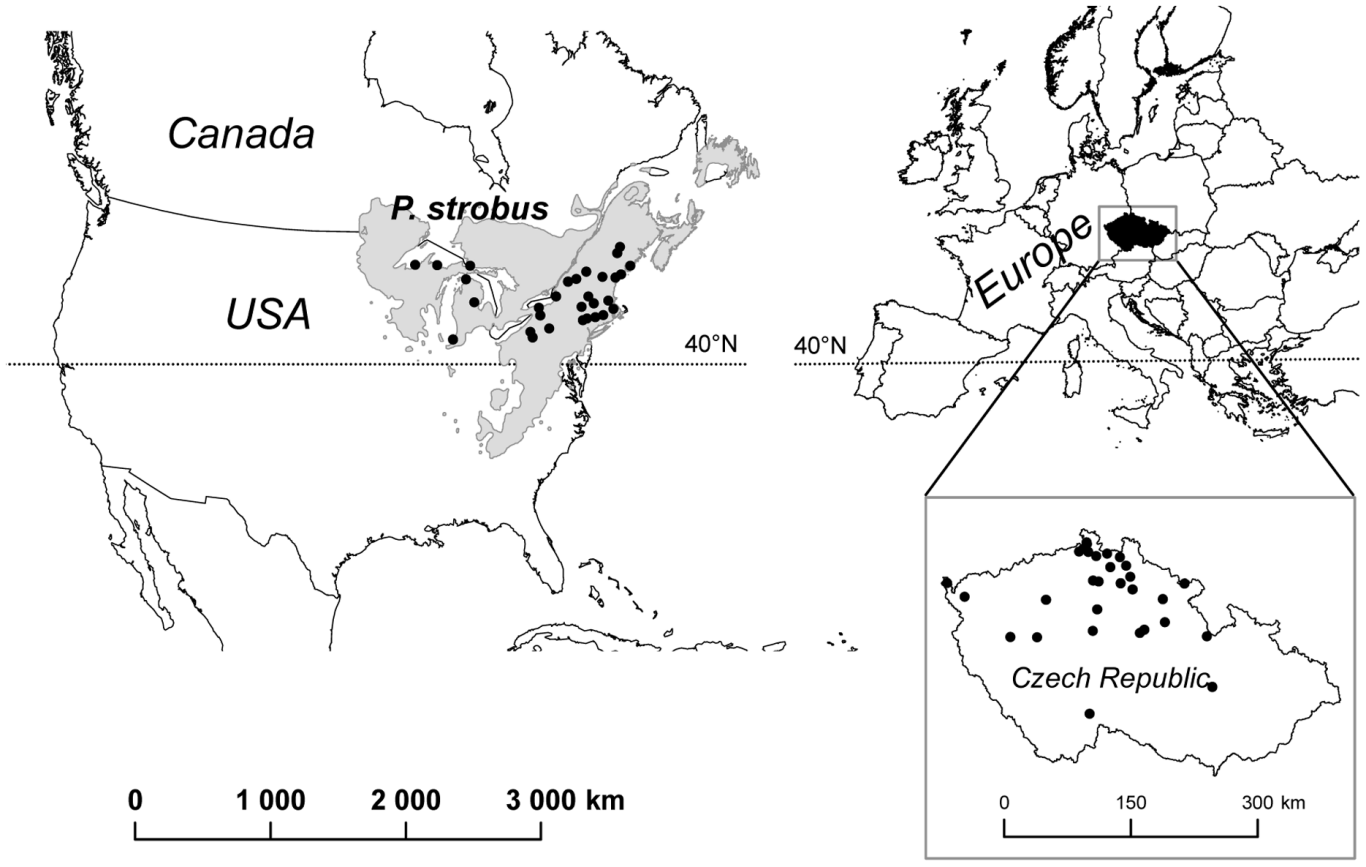
Geographical location of *Pinus strobus* study populations.

*Pinus strobus* L. (eastern white pine, also called Weymouth pine) is a conifer native to northeast North America [57], with its current distribution there shown in Fig. 1. In its native range, it is common in moist cool forests, near streams and rivers, and on rocky or sandy nutrient-poor and well drained sites [57]. The majority of *P. strobus* stands were deforested by the late 19^th^ century [58], and therefore, not many old-growth stands are left in its native area. However, because it is among the most rapidly growing northern conifers, it has been used extensively for reforestation projects [57].

*Pinus strobus* is highly invasive in some sandstone areas of the Czech Republic [53]. The first report of the introduction of a large amount of this species’ seeds intended for cultivation in the Czech Republic is from 1784 [54]. In the sandstone area, it was first cultivated in 1798 (Elbe River Sandstones – district Přední and Zadní Doubice). *Pinus strobus* was introduced into mixed conifer forests to increase community diversity and inhibit pest infestation in species-poor forests on nutrient poor sandy soils and sandy loams [54]. The ability of the species to regenerate easily was noticed from the beginning of its cultivation [54], [59], but its spread into surrounding forests was not spotted until the 1950s, when self-sown individuals were observed in the area of the Elbe River Sandstones [53]. A massive expansion took place in the same area in the 1990s, when the species entered the predominantly acidophilous pine (*Dicrano-Pinetum*) and oak-pine (*Vaccinio vitis-idaeae-Quercetum*) forests [53], which are typical plant communities of protected sandstone areas.

*Pinus strobus* is an outcrossing, wind-pollinated species whose winged seeds are dispersed by wind. In the adventive area, individual trees can be fertile at the age of twenty years, with the substantial production of seeds at the age of 50 years and older. Münzbergová *et al.* [60] found that younger, middle-aged trees (20–50 years old) contributed to the total seed set by only about 5% of all seeds and trees younger than 20 years have nearly no fertility. White pine produces many seedlings at all localities observed in sandstone areas and on sandy and sandy-loam soils [53], [61]. However, high mortality (50%) of seedlings younger than 5 years substantially reduces the number of individuals passing into the sapling stage. The mortality of saplings (5–20 years old), middle-aged (20–50 years old) and old (≥50 years old) trees is low (5%, 0.75% and 0.9%, respectively; [60]).

*Pinus strobus* possesses high seed and pollen dispersal capacity. Wind-dispersed seeds released from the upper canopy can travel several hundreds meters from the source tree, with the maximum recorded distance of a self-sown tree from the potential seed source found to be 757 m. However, the observed median distance for 50% of self-sown trees was only 52 m and only 1% of self-sown trees reached 502 m [62]. Some pine pollen has been reported to travel tens of kilometers [63], [64].

### Locality Selection and Sampling Procedures

#### General approach – determination and definition of age classes

In both large- and fine-scale studies, we aimed at obtaining samples from different phases of the invasion process. We thus sampled trees of several consecutive age classes which we define, and hereafter use, as follows: (1) “old trees” – trees 50 years old and older. While these trees are canopy trees (in the adventive range mostly planted); (2) “mature trees” – between 21 and 49 years old trees; (3) “saplings” – trees of age between 6 and 20 years; and (4) “seedlings” – trees not older than 5 years. Individual age classes were determined on the basis of a combination of mortality rate and fecundity estimated in a related study ([60], see Study species in Materials and Methods section). The age of old trees was either extracted from forest inventory data contained in forest management plans or estimated by counting whorls, which are formed annually [65]. Age determination based on whorl counting is precise up to the age of 50 years, where the Pearson correlation coefficient between the number of whorls (or branch scars) and number of rings above the root collar was found to be 0.979 in the Czech Republic [62]. The age of trees in the other age classes was estimated by whorl-counting, which is more precise in younger age classes.

#### Large-scale study

We sampled 60 localities, 30 in the native and 30 in the adventive range. Based on a comparative morphological study of seedlings, Musil [59], [66] stated that populations of *P. strobus* occurring in Europe were derived from a relatively small area near two important commercial ports (New York and Boston) along the eastern edge of its native range. Because the distribution of *P. strobus* in North America is quite broad [57], we sampled both in this area from which the species was most likely introduced and from other areas from which introduction was not as likely. A detailed list of individual locations is presented in Table 1. Localities were selected to be placed in close proximity to old-growth trees based on a survey of old-growth forests in the eastern USA done by Davis [67]. We sampled forests with the average age of the oldest trees about 83 years with the exception of one location where we sampled trees that were more than 300 years old (population A57). The sampling in the adventive range was done only within the Czech Republic (Fig. 1, Table 1), with an effort to sample all the areas in the country in which the species occurs. Samples were collected in July and August 2007 in the native range and from April 2007 to September 2008 in the adventive range.

**Table 1:** Summary of genetic diversity within 60 populations of *Pinus strobus* based on ten microsatellite loci.

Pop.	Locality	Latitude	Longitude	*N*	*A*	*H*_S_	*R*_S_	*H*_O_	*H*_E_	*f*(F_IS_)
**E1**	Doubice, Hrad	N50°53′26″	E14°24′58″	20	5.2	0.597	4.467	0.479	0.579	0.198*
**E2**	Doubice, Dravčí Skály	N50°54′25″	E14°24′25″	20	4.9	0.543	4.005	0.441	0.525	0.188*
E3	Hradec Králové	N50°10′13″	E15°52′34″	20	5.1	0.547	4.180	0.444	0.529	0.188*
E4	Klokočí	N50°36′20″	E15°12′46″	20	4.5	0.550	3.845	0.433	0.533	0.214*
**E5**	Brandýs-Stará Boleslav	N50°13′02″	E14°44′05″	20	5.5	0.569	4.478	0.433	0.550	0.240*
E6	Řečany nad Labem	N50°01′38″	E15°28′59″	20	5.2	0.614	4.362	0.500	0.594	0.185*
E7	Bílichov	N50°14′38″	E13°52′08″	21	5.2	0.543	4.302	0.372	0.524	0.314*
**E8**	Říčany	N49°59′10″	E14°42′25″	20	5.0	0.520	4.118	0.418	0.503	0.195*
E9	Jemčina	N49°06′30″	E14°50′04″	20	4.9	0.533	4.113	0.394	0.512	0.261*
E10	Adršpach	N50°36′08″	E16°07′33″	20	4.1	0.465	3.473	0.363	0.449	0.219*
E11	Studené	N50°04′20″	E16°35′19″	20	4.5	0.524	3.841	0.377	0.505	0.280*
E12	Blatce	N50°30′53″	E14°36′05″	19	5.0	0.544	4.274	0.459	0.526	0.156*
E13	Pařezská Lhota	N50°28′28″	E15°16′43″	20	6.0	0.517	4.689	0.385	0.499	0.255*
E14	Příhrazy	N50°31′35″	E15°03′54	20	5.0	0.546	4.175	0.437	0.528	0.198*
**E15**	Aš	N50°15′23″	E12°12′25″	20	5.4	0.585	4.443	0.452	0.565	0.227*
**E16**	Kynšperk nad Ohří	N50°08′32″	E12°31′42″	21	4.7	0.586	4.042	0.441	0.568	0.248*
**E17**	Cvikov	N50°46′31″	E14°36′02″	20	5.4	0.545	4.482	0.433	0.528	0.206*
E18	Liberec	N50°48′04″	E14°59′51″	21	5.7	0.563	4.523	0.467	0.547	0.171*
**E19**	Bezděz	N50°30′44″	E14°41′46″	20	5.2	0.570	4.315	0.489	0.551	0.141*
E20	Petrovice	N50°48′58″	E14°46′34″	20	5.4	0.573	4.338	0.473	0.555	0.174*
E21	Česká Kamenice	N50°48′36″	E14°27′35″	20	4.7	0.479	3.793	0.367	0.463	0.234*
**E22**	Stráž pod Ralskem, Ralsko	N50°40′53″	E14°51′36″	20	5.2	0.585	4.500	0.455	0.565	0.221*
**E23**	Jablonec nad Nisou	N50°43′06″	E15°07′03″	20	5.8	0.575	4.546	0.396	0.555	0.312*
E24	Dvůr Králové nad Labem	N50°24′45″	E15°47′50″	20	5.2	0.526	4.131	0.400	0.509	0.241*
E25	Zbiroh	N49°50′03″	E13°48′45″	20	5.1	0.560	4.075	0.472	0.544	0.157*
**E26**	Plzeň	N49°47′43″	E13°22′37″	20	5.3	0.596	4.443	0.482	0.577	0.191*
E27	Horní Štěpánov	N49°32′37″	E16°46′12″	20	4.8	0.557	4.070	0.399	0.537	0.284*
**E28**	Huntířov, Bynovec	N50°48′01″	E14°18′08″	20	5.1	0.560	4.121	0.455	0.543	0.187*
**E29**	Srbská Kamenice	N50°50′01″	E14°22′08″	20	5.6	0.527	4.589	0.446	0.511	0.154*
**E30**	Sopřeč	N50°04′26″	E15°32′54″	20	4.8	0.547	4.075	0.429	0.528	0.216*
A31	NY, Millbrook	N41°47′38″	W73°43′59″	20	5.0	0.591	4.145	0.461	0.572	0.221*
A32	NY, New Paltz	N41°45′42″	W74°09′46″	20	5.2	0.476	4.097	0.422	0.462	0.114*
A33	NY, Albany	N42°35′27″	W74°00′28″	20	5.3	0.546	4.378	0.368	0.526	0.326*
A34	PA, Wellsboro	N41°41′48″	W77°27′18″	20	5.5	0.552	4.381	0.422	0.534	0.236*
A35	PA, Clarington	N41°20′27″	W79°08′00″	20	5.6	0.522	4.409	0.352	0.504	0.326*
A36	PA, Warren	N41°42′04″	W79°14′56″	20	4.4	0.516	3.782	0.433	0.497	0.161*
A37	NY, Castile	N42°36′28″	W78°01′30″	20	5.2	0.587	4.386	0.442	0.568	0.246*
A38	NY, Bergen	N43°05′24″	W78°02′00″	20	5.3	0.574	4.335	0.507	0.557	0.117*
A39	NY, Pulaski	N43°33′43″	W76°11′40″	20	5.9	0.526	4.515	0.400	0.509	0.240*
A40	NY, Tupper Lake	N44°18′41″	W74°43′16″	20	5.9	0.516	4.478	0.411	0.499	0.203*
A41	NY, Wilmington	N44°21′46″	W73°50′32″	20	5.7	0.559	4.575	0.468	0.540	0.162*
A42	VT, Johnson	N44°39′11″	W72°43′38″	20	5.8	0.577	4.673	0.417	0.557	0.277*
A43	NH, Bartlett	N44°04′30″	W71°18′17″	20	5.4	0.499	4.184	0.444	0.485	0.111*
A44	ME, Freeport	N43°49′22″	W70°05′36″	20	5.6	0.537	4.316	0.396	0.519	0.262*
A45	ME, Guilford	N45°15′53″	W69°16′55″	20	5.4	0.560	4.422	0.405	0.542	0.277*
A46	ME, Millinocket	N45°35′47″	W68°48′51″	20	5.2	0.548	4.154	0.467	0.532	0.149*
A47	ME, Seal Cove	N44°16′40″	W68°22′45″	20	4.1	0.484	3.376	0.468	0.471	0.033
A48	ME, New Harbor	N43°54′48″	W69°29′12″	20	5.5	0.567	4.459	0.461	0.548	0.187*
A49	MA, Chelmsford	N42°33′31″	W71°20′55″	20	5.3	0.536	4.359	0.473	0.521	0.117*
A50	MA, Taunton	N41°58′42″	W71°04′52″	20	5.5	0.548	4.311	0.466	0.532	0.150*
A51	VT, Manchester	N43°07′41″	W73°07′05″	20	5.9	0.559	4.659	0.487	0.543	0.128*
A52	MA, Shelburne Falls	N42°37′30″	W72°46′13″	20	5.8	0.551	4.653	0.453	0.533	0.179*
A53	CT, Mansfield Center	N41°45′60″	W72°10′47″	20	6.3	0.600	4.898	0.478	0.582	0.203*
A54	CT, Burlington	N41°46′14″	W72°57′13″	20	5.0	0.497	4.070	0.443	0.483	0.109*
A55	MI, Alger	N44°04′04″	W84°10′58″	20	5.2	0.559	4.306	0.489	0.543	0.125*
A56	MI, Pellston	N45°32′54″	W84°41′58″	20	5.2	0.602	4.458	0.433	0.581	0.280*
A57	MI, White Pine	N46°44′89″	W89°42′48″	20	6.2	0.592	4.843	0.453	0.572	0.235*
A58	MI, Marquette	N46°37′21″	W87°28′20″	20	5.5	0.56	4.490	0.486	0.543	0.132*
A59	MI, Sault Ste. Marie	N46°21′23″	W84°08′22″	20	6.1	0.536	4.884	0.432	0.518	0.195*
A60	MI, Bridgman	N41°54′17″	W86°36′25″	12	4.6	0.501	4.302	0.367	0.475	0.268*
Mean					5.3	0.549	4.302	0.437	0.531	0.204
SD					0.5	0.033	0.300	0.038	0.032	0.061

Pop. = Population – the first letter of each population name refers to the continent in which the population was found, i.e. E = Europe (Czech Republic) and A = North America; populations in bold were classified as invasive within the adventive range; GPS coordinates of sampling sites are in WGS84; *N* – number of individuals sampled from each population; *A* – average number of alleles per locus; *H*_S_ – mean gene diversity over all loci; *R*_S_ – mean allelic richness; *H*_O_ – observed heterozygosity; *H*_E_ – expected heterozygosity; *f*(F_IS_) – inbreeding coefficient according to Weir and Cockerham [77]. Populations deviating from Hardy-Weinberg equilibrium at P<0.05 are marked by asterisk. SD – standard deviation. The fine scale studies were performed at localities E1, E22, E28 and E30.

We collected several needles from each of 20 plants at each locality wherever possible. Some localities, however, had such small numbers of trees that we could only sample fewer individuals there (Table 1). At each locality, we aimed at sampling trees of all age cohorts where it was possible. Because various authors [68], [69] have detected a significant fine-scale genetic structure in native American populations at 15 m intervals, we sampled at this interval. Since little is known about the fine-scale genetic structure of *P. strobus* populations in Central Europe, we sampled plants at the same interval in the adventive range to ensure sampling consistency. Within a population, we would locate individuals to be sampled by creating a 60 by 45 m rectangular grid with the gridlines spaced at 15 m intervals, and then collecting from those trees located at the intersections of the gridlines. If no *P. strobus* tree was located at one of these points, we would use the plant nearest to the point but not further than 1 m away in cases of trees not older than 10 years, 2.5 m away in cases of trees not older than 50 years, and 5 m away in cases of trees older than 50 years. If the resulting area surrounding a point did not contain any usable tree, we skipped that point and extended our grid by 15 m to generate a replacement point. Where possible, we would continue sampling until we collected samples from twenty trees. In total we collected 1194 samples.

In the Czech populations, we later used the number of points in our grid not occupied by trees for discrimination between invasive and naturalized populations (sensu Richardson *et al.* [56]). A population was considered invasive if more than 90% of points contained a self-sown individual; otherwise it would be classified as naturalized (see Table 1). Populations classified as invasive clearly differed also visually from naturalized ones in that the former would include seedlings and saplings nearly completely covering available space, while the naturalized populations were predominantly composed of old trees with only a few self-sown individuals. For each population in the adventive range we also collected data on elevation above sea level, long term average annual precipitation and air temperature [70] and soil types (taken from National geoportal INSPIRE, http://geoportal.gov.cz).

#### Fine-scale study

We chose four localities in different parts of the Czech Republic, all of them being highly affected by the invasion of *Pinus strobus.* The four sampling areas were (see Table 1): (1) Bynovec – locality E28, (2) Hrad – locality E1, (3) Ralsko – locality E22 and (4) Sopřeč – locality E30 (for details on individual localities see Table 1). To obtain samples from different phases of the invasion process, we sampled trees belonging to the consecutive age classes as described above, i.e. old trees, mature trees, saplings, and seedlings.

In each of the four populations, we intended to collect 50 samples of each age class from regularly distributed plots, if possible. To ensure sampling consistency, we used the same sampling strategy at all localities. At each locality, we sampled all old trees because their number was always limited. Afterwards, in the core of each stand, we created a 40 by 100 m grid with gridlines spaced at 10 m intervals. We then took one sample of each age class within each square (except for old trees), i.e., 50 samples from each age class were taken. Saplings were originally divided into old (50 samples) and young saplings (50 samples) that were later merged due to our inability to precisely define these groups on the basis of mortality and fecundity parameters. In most cases, we were unable to collect all 250 samples at each locality, in particular due to the lack of old trees. In total, we collected 954 samples and recorded the spatial coordinates of each. The positions of all trees were recorded using a post-processing DGPS technique (GPS Trimble Pathfinder Pro XRS). All GPS positions were corrected to achieve sub-meter accuracy using data from two-reference base stations located in distance of up to 50 km (data provided by Czech office for surveying mapping and cadastre; http://www.cuzk.cz).

### Molecular Methods

#### DNA extraction

The needles were stored in CTAB. DNA from the *P. strobus* samples was isolated as described in Štorchová *et al.* [71], with only needles crushed in liquid nitrogen. The quality and yield of isolated DNA was checked on agarose gels and then precisely measured for DNA concentration using a biophotometer (Eppendorf, Germany). All samples were then diluted to a 15–30 ng/µl concentration, which is suitable for PCR with labeled microsatellite primers.

#### Microsatellite analysis

We analysed genetic variation at ten nuclear microsatellite loci in both the large-scale genetic diversity study (i.e., 1194 samples) and the fine-scale genetic diversity study (i.e., 954 samples). These loci were RPS1b, RPS2, RPS12, RPS25b, RPS34b, RPS39, RPS50, RPS84, RPS118b and RPS127 [72], [73]. DNA amplification was carried out in three multiplex PCRs (multiplex 1: RPS1b, RPS2, RPS12 and RPS39; multiplex 2: RPS25b, RPS34b, RPS50 and RPS127; multiplex 3: RPS84 and RPS118b). DNA was amplified using the QIAGEN Multiplex PCR kit (QIAGEN, Germany) in a total reaction volume of 5 µl of PCR mix plus 5 µl of mineral oil to avoid PCR mix evaporation, containing 15–30 ng of DNA, 0.1–0.5 µM of each primer, and 2.5 µl of Master Mix (QIAGEN). To improve the quality of the PCR product, we added Q-solution (QIAGEN) and 4 mM MgCl_2_ in Multiplex 3. PCR amplifications were conducted in a Mastercycler (Eppendorf) under the following conditions: an initial denaturation step of 15 min at 95°C followed by 40 cycles of 30 s at 94°C, 90 s at 60°C, 60 s at 72°C and a final extension of 10 min at 72°C.

PCR products were electrophoresed in an ABI PRISM 3130 sequencer (Applied Biosystems, USA). One microlitre of PCR product was mixed with 0.2 µl of GeneScan-500 LIZ (Applied Biosystems) and 12 µl of formamide (Applied Biosystems). Allele sizes were determined using GeneMapper version 4.0 software (Applied Biosystems). The raw data are available by request from the authors. An individual was declared null (nonamplifying at a locus) and treated as missing data after at least two amplification failures. The diagnostic results using MICRO-CHECKER [74] found no evidence of stuttering or large allele drop-out for any of the loci. However, the potential occurrence of null alleles was detected in loci RPS25b and RPS34b, which mostly corresponded to the deviation from Hardy–Weinberg equilibrium.

### Statistical Analysis – Large-scale Study

#### Genetic diversity measures

Summary data for SSR loci, including the average number of alleles per locus (*A*), mean gene diversity overall loci (*H*_S_), mean allelic richness (*R*_S_) (here allelic richness is a metric that uses a rarefaction index to take into account differences in sample size [75], [76]) and Weir & Cockerham’s parameter *f*(*F*_IS_) [77], a measure of deviation from random mating within a population, were calculated using Fstat [76]. Observed (*H*_O_) and expected (*H*_E_) heterozygosities were calculated using the program Arlequin [78], and deviation from the Hardy-Weinberg equilibrium was determined on the basis of 10,000 permutations in Fstat. Sequential Bonferroni corrections were applied to adjust *P* value according to Rice [79].

Weir & Cockerham’s [77] estimates of Wright’s [80] *F* statistics were generated for all loci. Significant deviations from the null expectation of *F*  = 0 were determined by 5000 bootstrap replicates using Fstat. In the bootstrap analysis, *F* (corresponding to Wright’s *F*_IT_) was estimated by alleles permutated across the entire dataset, *f*(*F*_IS_) was estimated by the permutation of alleles within populations, and *θ*(*F*_ST_) was estimated by the permutation of alleles among populations. The allele frequency-based *F*_ST_ was used rather than the allele size-based *R*_ST_ of Slatkin [81], which is derived specifically under the assumptions of the generalized stepwise-mutation model (SMM). *R*_ST_ and *F*_ST_ values are not expected to differ greatly for short-term differentiation of populations within species [81], but *F*_ST_-based estimates of differentiation are considered more reliable when fewer than 20 loci are used [82].

Comparisons of genetic diversity parameters between groups (i.e. native North American and non-native European populations or invasive and naturalized non-native populations) were performed with Fstat with 10,000 permutations.

We used the Mantel test to assess the model of isolation-by-distance using genetic distance for pairs of populations [83] and geographic distance among these populations (calculated using Fstat). To test differences in allele frequencies, we used the exact test for population differentiation [84]. This analysis uses a contingency table approach (Fisher’s R × C-test) [85] to determine whether significant differences in allele frequencies exist among groups of individuals (calculated using TFPGA, http://www.marksgeneticsoftware.net).

#### Bottleneck detection

We tested for an excess of heterozygosity, which reveals the loss of rare alleles in a bottlenecked population relative to that expected under the mutation-drift equilibrium (neutrality) for an observed level of heterozygosity [86], using microsatellite frequency data. In a population which has recently been reduced in size, both the number of alleles (allelic diversity) and heterozygosity would likely be reduced. However, allelic diversity would be reduced faster than heterozygosity, resulting in deficiency in the observed number of alleles relative to the number of alleles expected from the observed heterozygosity, providing the basis for this test. Microsatellite data were processed using the BOTTLENECK program [86], [87]. We considered the two-phase model (TPM), which is probably closer to the true mode of mutation at most microsatellite loci [87]. The proportion of alleles attributed to a stepwise-mutation model (SMM) under TPM was set to 85%, with a variance of 5. Ten thousand iterations were run. The one-tailed Wilcoxon signed rank test for heterozygote excess was applied as a test of significance [86], [87], and the distribution of allele frequencies was tested against the L-shaped distribution, as expected under the mutation-drift equilibrium [88].

#### Population structure and identification of sources

**Figure 2:**
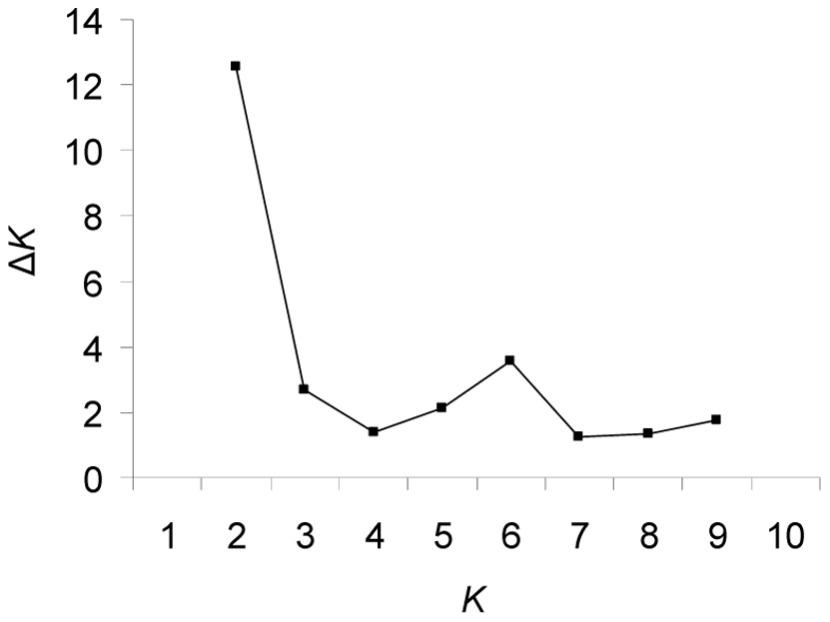
Estimation of the number of genetic clusters using Bayesian assignment technique. Second-order rate of change in the probability between successive runs (Δ*K*) as a function of *K* (number of clusters).

We used Structure version 2.3.3 software [89] to estimate the number of genetic clusters (*K*) and to fractionally assign individuals sampled in North America and Europe to the inferred groups. We applied the model which allows population admixture with prior population information [90], and correlated allele frequency [89]. The model with prior population information was used because of a weak population structure [90]. The number of clusters (*K*) was set at each value from one through ten, and the simulation was run ten times at each *K* value to confirm the repeatability of the results. Each run comprised a burn-in period of 150,000, followed by 300,000 Markov chain Monte Carlo (MCMC) steps. Of the various ways to estimate the ‘true’ number of genetic clusters using Bayesian assignment techniques [89], [91], [92], we used the one [91] in which the quantity Δ*K* is employed as an *ad hoc* estimator of the second order rate of change of the lnP(K|X) to furnish an initial estimate of *K* (see Fig. 2). Per Evanno *et al.* [91], our modelling demonstrated that the peak (modal) value(s) of Δ*K* were good estimates of *K* in simulations with a range of known population sizes and types and numbers of loci (but see [92]). The output of structure analyses was visualized using CLUMPP [93] and DISTRUCT [94] software.

#### Ecological factors

The effects of ecological factors (elevation above sea level, average annual precipitation and temperature and soil types) on *P. strobus* population regeneration ability were analysed by Logit models implemented in the Generalized Linear/Nonlinear Models module of the software package STATISTICA 9.1 (http://www.statsoft.com).

### Statistical Analysis – Fine-scale Study

#### Genetic diversity measures

Genetic diversity parameters were calculated as described in the large-scale study (see above). Comparisons of genetic diversity parameters among populations were performed with Fstat with 10,000 permutations. Pairwise *F*_ST_ values were used to compare differences in gene frequencies among different age classes within each locality with Fstat, and a Bonferroni correction was used to adjust observed *P* values for multiple comparisons.

Genetic variation at the level of populations and age classes was investigated with a nested analysis of molecular variance (age classes nested within populations; AMOVA – [78]). Levels of significance were determined by computing 1,000 random permutation replicates.

#### Spatial Genetic Structure (SGS)

Fine-scale genetic structure was assessed by spatial autocorrelation analysis of genetic relatedness between pairs of individuals as described in [95], [96] and defined as a ratio of probabilities of identity by descent. Assuming migration-drift equilibrium, the impact of mutations at small spatial scales is negligible relative to that of gene flow, and the ratio of probabilities of identity in state is similar to that of probabilities of identity by descent [97]. A pairwise estimate of genetic correlation, *f_ij_* (co-ancestry coefficient; sensu Kalisz *et al.* [96]) measures the frequency of allele sharing between two individuals relative to average frequency of allele sharing based on the frequencies of the alleles in the sampled population [98]. Co-ancestry (*f_ij_*) was estimated between all pairs of individuals within each age class following the methods of Loiselle *et al.* [95] and Kalisz *et al.* [96]. To analyse the relationships between pairwise physical distances and co-ancestry (*f_ij_*) coefficient, we calculated *f_ij_* for 5 m distance intervals up to 100 m. Mean values of *f_ij_* were obtained for distance intervals of 5 m by averaging over all pairs of individuals located within that interval. Spatial autocorrelation analysis was also computed for all individuals within each population irrespectively of age class and among age classes within each population to test whether the spatial structure of individual age classes were dependent on each other. When *f_ij_  = *0, there is no significant correlation among individuals at the spatial scale of interest; when *f_ij_* >0, individuals in a given distance class are more closely related than expected by chance; and when *f_ij_* <0, individuals within a given distance class are less related than expected by chance with respect to the local population. Co-ancestry coefficients were computed using SPAGeDi software [99].

We also evaluated the strength of isolation by distance using *Sp* statistics, calculated as –*b*/(1–*F*_(1)_), where *F*_(1)_ is the *f_ij_* for the first distance class. *F*_(1)_ can be considered an approximation of the kinship between pairs of neighbours, provided the first distance class contains enough pair of individuals to obtain a reasonably precise *F*_(1)_ value [42].

## Results

### Large-scale Study

#### Genetic diversity, population structure and regional differentiation

While tests for genotypic linkage disequilibrium were all non-significant after applying sequential Bonferroni correction, tests of deviation from the Hardy-Weinberg equilibrium (Table 1) were all significant with the exception of locality A47. We identified 178 alleles at ten microsatellite loci, with an average of 17.8 alleles per locus across all populations. Populations in the adventive range had a very similar average number of alleles per locus and population to that of native ones (*A*  = 5.1 vs. 5.4) (Table 1). No genetic diversity measures differed significantly between the native and adventive ranges, with the exception of allelic richness (*R*_S_), where the difference was marginally significant (Table 2), suggesting slightly higher allelic richness in the adventive range. These data show that the genetic diversity found within the relatively small adventive range, surprisingly, equals the diversity found within the sampled area in the native range, which is about thirty times larger (Fig. 1). Only 135 alleles were observed in the adventive range compared to 159 alleles found in native populations. The difference is due to the higher number of rare alleles in the native range that are absent in the adventive range. Therefore, there were more unique genotypes (i.e. combinations of alleles at individual loci) present in the native range (31.3% of individuals had at least one unique genotype at a locus compared to 26.4% in the adventive range). The value of *f*(*F*_IS_) was lowest in native population A47 (0.033), and highest in native populations A33 and A35 (0.326), which indicates the existence both of populations in Hardy-Weinberg equilibrium and of populations showing excesses of homozygotes, suggesting some level of inbreeding (Table 1).

**Table 2:** Comparison of population genetic parameters between native and adventive range.

Diversity measure	Native	Adventive	*P*
*R*_S_	4.227	4.377	0.052
*H*_O_	0.432	0.440	0.418
*H*_S_	0.550	0.543	0.452
*f*(*F*_IS_)	0.215	0.191	0.129
*θ*(*F*_ST_)	0.025	0.030	0.513

Statistical comparison of allelic richness (*R*_S_), observed heterozygosity (*H*_O_), gene diversity (*H*_S_), inbreeding coefficient *f*(*F*_IS_), and levels of differentiation among populations *θ*(*F*_ST_) for the native and adventive ranges of *Pinus strobus*. Probability values for differences between the native and the adventive range are given for two-sided *t*-test, after 10,000 permutations. The analysis was performed using Fstat software.

To investigate the pattern of inbreeding and population differentiation further, we analysed genetic variation and structure for individual microsatellite loci and over all loci separately for native and non-native populations. The mean value of *f*(*F*_IS_) was quite high and significantly different from zero in most cases within both ranges (Table 3), with a tendency of populations in the adventive range to be less inbred than native populations. Significant differentiation among populations in both ranges was detected, with slightly higher *θ*(*F*_ST_) values reached by populations in the adventive range (Table 3). However, neither *f*(*F*_IS_) nor *θ*(*F*_ST_) differed significantly between the native and adventive ranges (Table 2). When we compared allele frequencies between the native and adventive ranges, we obtained significant differences at all individual loci (data not shown) as well as over all loci (χ^2^ = 269.26, d.f.  = 20, p<10^–4^).

**Table 3:** Genetic variation and structure for 10 polymorphic loci identified in 60 *Pinus strobus* populations.

	Total			Native range			Adventive range		
Locus	*F*(*F*_IT_)	*f*(*F*_IS_)	*θ*(*F*_ST_)	*F*(*F*_IT_)	*f*(*F*_IS_)	*θ*(*F*_ST_)	*F*(*F*_IT_)	*f*(*F*_IS_)	*θ*(*F*_ST_)
RPS1b	**0.089**	**0.078**	**0.013**	**0.104**	**0.103**	0.002	**0.074**	0.055	**0.020**
RPS25b	**0.615**	**0.589**	**0.064**	**0.624**	**0.600**	**0.062**	**0.605**	**0.578**	**0.062**
RPS39	**0.261**	**0.250**	**0.014**	**0.276**	**0.271**	0.006	**0.253**	**0.236**	**0.023**
RPS84	**0.039**	0.013	**0.026**	0.041	0.032	**0.009**	0.016	0.001	**0.015**
RPS2	0.002	–0.021	**0.022**	–0.032	–0.040	**0.008**	0.031	0.011	**0.020**
RPS12	**0.197**	**0.175**	**0.027**	**0.168**	**0.154**	**0.015**	**0.216**	**0.196**	**0.025**
RPS34b	**0.318**	**0.286**	**0.045**	**0.394**	**0.369**	**0.039**	**0.219**	**0.190**	**0.035**
RPS50	**0.059**	**0.024**	**0.036**	**0.028**	0.003	**0.025**	**0.071**	**0.045**	**0.027**
RPS118b	**0.322**	**0.306**	**0.023**	**0.409**	**0.402**	**0.012**	**0.228**	**0.208**	**0.024**
RPS127	**0.093**	**0.063**	**0.031**	0.055	0.019	**0.037**	**0.130**	**0.112**	**0.020**
Over all loci	**0.228**	**0.202**	**0.033**	**0.234**	**0.215**	**0.025**	**0.215**	**0.191**	**0.030**

*F*, *f*, *θ* = Weir and Cockerham’s estimates of Wright’s *F* statistics (*F*_IT_, *F*_IS_ and *F*_ST_, respectively), which represent deviations from Hardy-Weinberg expectations over all populations, deviations within individual populations and the proportion of total genetic diversity partitioned among populations. Significant deviations (P<0.05) from the null expectation of *F*  = 0 are indicated in bold.

There was no consistent association between genetic and geographic distances in both the native and the adventive range, as indicated by the Mantel tests (*r* = –0.003, *R* ^2^ = 0.001, *P*  = 0.947 and *r* = –0.081, *R* ^2^ = 0.66, *P*  = 0.088, respectively).

#### Bottleneck

We found no evidence for an excess of heterozygotes in any of the populations tested using the TMP mutation model. Allele frequencies followed an L-shaped distribution; that is, no skews of allele frequencies towards intermediate values were observed in any of the populations, with such a finding expected from a non-bottlenecked population at mutation-drift equilibrium. This indicates that the populations in the adventive range were not exposed to strong bottlenecks in their recent history (data not shown). We note, however, that the heterozygote excess/deficit test has been known to miss bottlenecks, as it was accurate in only 50–75% of test studies [88].

#### Population structure and identification of sources

**Figure 3:**
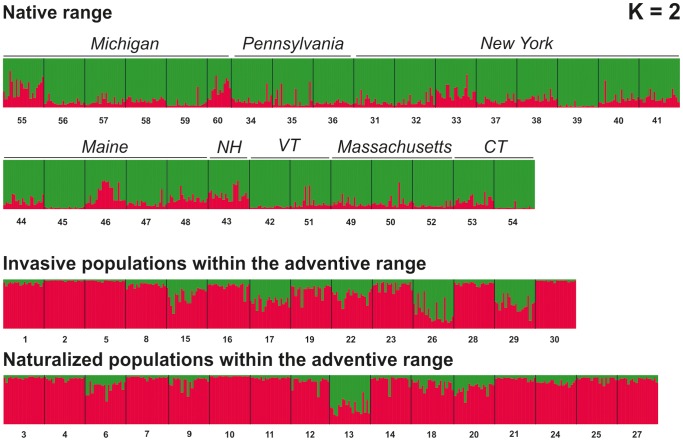
Results of assignment test. Percentage assignment of each North American and European individual (represented by vertical bars) to each of the two clusters (represented by different colors) inferred by the program Structure. Site codes (see Table 1) indicate the geographical location of individuals along the *x*-axis.

In the Bayesian clustering analysis, Δ*K* indicated that two clusters best explained the genetic structuring of *P. strobus* populations (Fig. 2). Assignments to more than two clusters did not provide unequivocal and easily interpretable results, as many clusters were mixed together, indicating that quite a high amount of genetic material from genetically very diverse population(s) was introduced from North America to Europe. Hence, populations were assigned to two clusters corresponding mainly to the native and the adventive ranges (Fig. 3).

#### Regeneration ability of *Pinus strobus* within the adventive range

The regeneration ability (in terms of the categorization scheme described in Materials and Methods) of the studied Czech populations was not affected by elevation above sea level, precipitation, temperature, or soil types, as indicated by Logit models (data not shown).

Invasive and naturalized populations differed in gene diversity (*H*_S_), although the differences were very weak (Table 4). Otherwise, they did not differ significantly in allelic richness (*R*_S_), observed heterozygosity (*H*_O_), inbreeding *f*(*F*_IS_) or fixation *θ*(*F*_ST_) coefficients (Table 4). Furthermore, invasive and naturalized populations differed significantly in allele frequencies (χ^2^ = 65.33, d.f.  = 20, p<10^–4^).

**Table 4:** Comparison of population genetic parameters between invasive and naturalized populations in the adventive range.

Diversity measure	Invasive	Naturalized	*P*
*R*_S_	4.330	4.137	0.073
*H*_O_	0.447	0.423	0.081
*H*_S_	0.565	0.540	**0.034**
*F*_IS_	0.210	0.217	0.734
*F*_ST_	0.019	0.032	0.256

Statistical comparison of allelic richness (*R*_S_), observed heterozygosity (*H*_O_), gene diversity (*H*_S_), inbreeding coefficient (*F*_IS_), and levels of differentiation among populations (*F*_ST_) for the invasive (N  = 14) and naturalized populations (N  = 16) of *Pinus strobus* within its adventive range in Europe. Probability values for differences between invasive and naturalized populations are given for two-sided *t*-test, after 10,000 permutations. The analysis was performed using Fstat software. Significant differences are indicated in bold.

### Fine-scale Study

#### Genetic diversity of the invasive populations

A total of 954 individuals from four localities were analysed across four age classes (Table 1). All 10 microsatellite loci analysed were polymorphic. While our comparison of genetic diversity parameters among four localities of *Pinus strobus* (two-sided *t*-test, after 10,000 permutations; the analysis performed using Fstat) showed similarities for allelic richness (*R*_S_, *p*  = 0.796), gene diversity (*H*_S_, *p*  = 0.130) and fixation indices [*θ*(*F*_ST_)_,_ *p*  = 0.977], there was a significant difference in observed heterozygosity (*H*_O_) and inbreeding coefficient [*f*(*F*_IS_)_,_ *p*  = 0.004 and *p*  = 0.005, respectively]. Especially the locality Hrad showed a lower level of heterozygosity and consequently higher inbreeding (Table 5).

**Table 5:** Summary of population genetic characteristics of *Pinus strobus* populations at which fine scale genetic study was performed.

Locality	Age class	No.	*A*	*R*_S_	*H*_O_	*H*_S_	*f*(*F*_IS_)
**Bynovec**	Old trees	50	6.0	4.794	0.468	0.569	0.179
	Mature trees	50	6.1	4.813	0.482	0.568	0.152
	Saplings	100	6.5	4.748	0.480	0.572	0.163
	Seedlings	50	5.7	4.738	0.473	0.568	0.169
	Overall	250	6.1	4.773	0.476	0.569	0.166
**Hrad**	Old trees	40	6.8	5.303	0.463	0.564	0.181
	Mature trees	50	7.6	5.340	0.434	0.565	0.234
	Saplings	100	6.5	4.790	0.412	0.565	0.272
	Seedlings	50	5.4	4.522	0.403	0.536	0.249
	Overall	240	6.6	4.989	0.428	0.558	0.234
**Ralsko**	Old trees	22	5.9	5.454	0.468	0.587	0.208
	Mature trees	49	5.7	4.580	0.474	0.557	0.150
	Saplings	100	6.5	4.694	0.435	0.552	0.212
	Seedlings	50	5.3	4.477	0.447	0.536	0.168
	Overall	221	5.9	4.801	0.456	0.558	0.185
**Sopřeč**	Old trees	43	5.6	4.726	0.472	0.567	0.170
	Mature trees	50	6.6	5.212	0.448	0.559	0.200
	Saplings	100	6.8	4.738	0.461	0.553	0.168
	Seedlings	50	6.1	4.750	0.455	0.563	0.194
	Overall	243	6.3	4.856	0.459	0.561	0.183

Number of individuals of *Pinus strobus* collected and analysed (No.) at different localities in the Czech Republic, average number of alleles per locus (*A*), allelic richness (*R*_S_), observed heterozygosity (*H*_O_), gene diversity (*H*_S_) and inbreeding coefficient *f*(*F*_IS_) according to [77] for 10 microsatellite loci of four recognized age classes – “old trees” (50 years old and older), “mature trees” (between 21 and 49 years old), “saplings” (between 6 and 20 years old) and “seedlings” (trees not older than 5 years) computed by Arlequin and Fstat. All populations significantly deviated from the Hardy-Weinberg equilibrium calculated using 50,000 permutations in Fstat.

Regression analyses of parameters of genetic diversity, i.e. allelic richness (*R*_S_), observed heterozygosity (*H*_O_), gene diversity (*H_S_*) and inbreeding coefficient (*F*_IS_) over time, i.e. from old trees to seedlings, with the dependent factor being the genetic diversity parameter and the independent one being time, were not statistically significant (data not shown).

A hierarchical analysis of molecular variance (AMOVA) revealed that a majority of the genetic diversity was partitioned within individuals (79.76%, P<10^–6^). Only 19.25% (P<10^–6^) was partitioned among individuals within age classes and 0.007% (P  = 0.446) was partitioned among age classes within localities. The remaining 0.97% (P<10^–6^) of the variation was due to differences among localities.

Pairwise *F*_ST_ values revealed that allele frequencies were not significantly different among individual age classes at all localities but one (Hrad). Allele frequencies at the locality Hrad significantly differed between seedlings and mature trees (*P*  = 0.01), seedlings and old trees (*P*  = 0.05) and between saplings and mature trees (*P*  = 0.05) (data not shown).

#### Spatial Genetic Structure (SGS)

**Figure 4:**
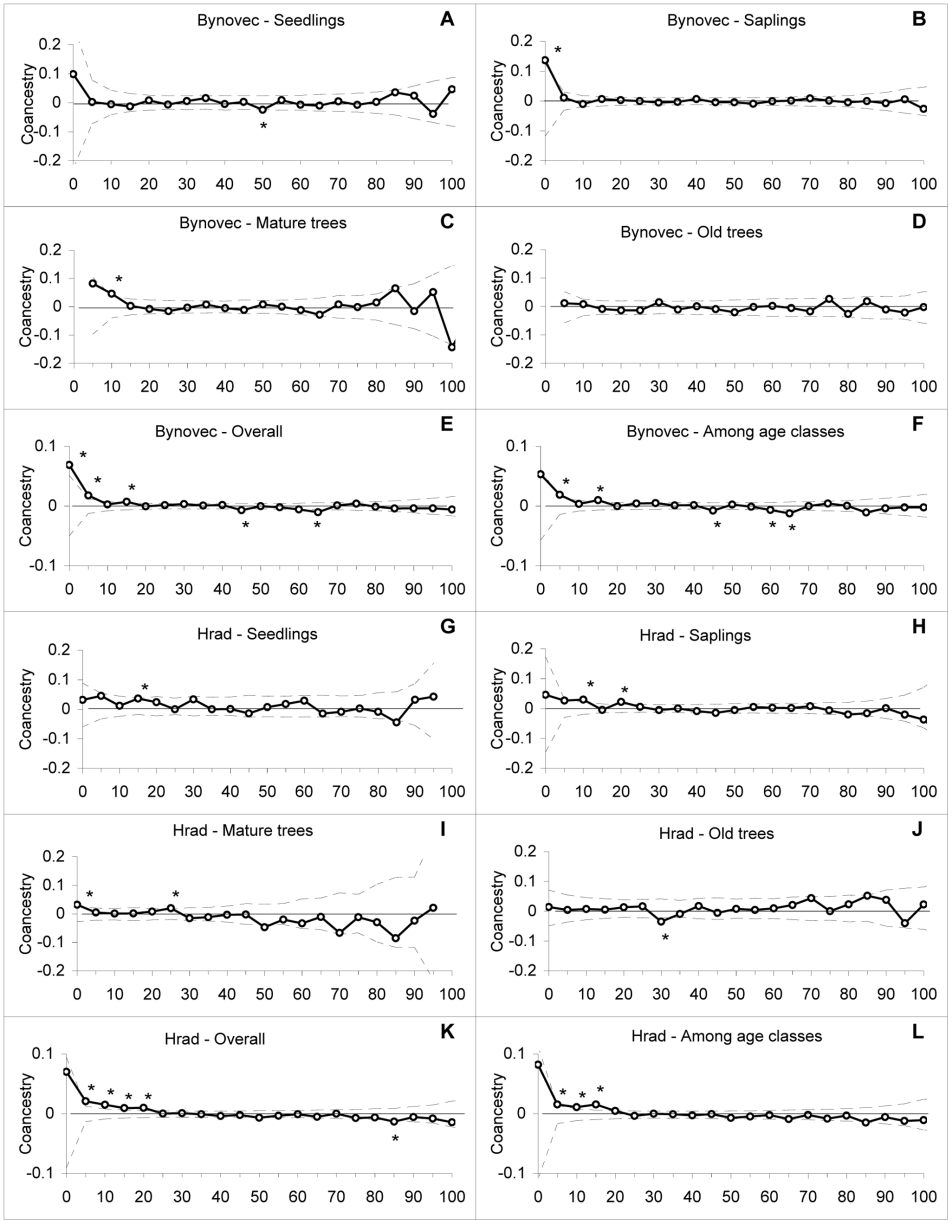
Correlograms of *f_ij_* coefficients for the localities Bynovec and Hrad. Correlograms of *f_ij_* coefficients for the locality Bynovec within (A) seedlings, (B) saplings, (C) mature trees, (D) old trees, (E) overall data and (F) among all age classes; for the locality Hrad within (G) seedlings, (H) saplings, (I) mature trees, (J) old trees, (K) overall data and (L) among all age classes. The solid line plots the observed data, and the dotted lines indicate the 95% confidence interval deduced from 10,000 permutations of individual multilocus genotypes within each age class, overall age classes or among all age classes. Values with asterisks (*) above and below the confidence envelopes indicate a greater or lesser genetic structure among individuals at different distances than expected of individuals chosen at random (*P<*0.005).

**Figure 5:**
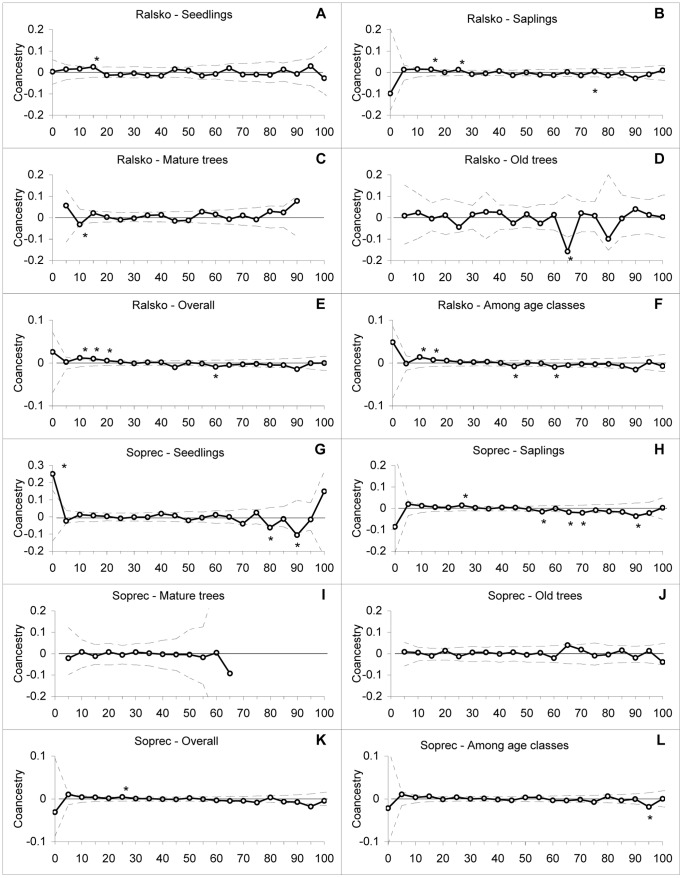
Correlograms of *f_ij_* coefficients for the localities Ralsko and Sopřeč. Correlograms of *f_ij_* coefficients for the locality Ralsko within (A) seedlings, (B) saplings, (C) mature trees, (D) old trees, (E) overall data and (F) among all age classes; for the locality Sopřeč within (G) seedlings, (H) saplings, (I) mature trees, (J) old trees, (K) overall data and (L) among all age classes. The solid line plots the observed data, and the dotted lines indicate the 95% confidence interval deduced from 10,000 permutations of individual multilocus genotypes within each age class, overall age classes or among all age classes. Values with asterisks (*) above and below the confidence envelopes indicate a greater or lesser genetic structure among individuals at different distances than expected of individuals chosen at random (*P<*0.005).

Our results indicated differences in SGS for different age classes within the investigated populations (Figs. 4, 5). In old trees, i.e. trees planted by foresters that founded the populations more than 100 years ago, we found no positive autocorrelation in any distance class or in any population. Mature trees showed positive autocorrelations at 10 and 25 m distances at the localities Bynovec and Hrad, respectively (Figs. 4C, I). Saplings were significantly structured in all tested localities (Figs. 4, 5, Table 6). In most cases, positive autocorrelations were in distance classes between 5 and 25 m. Populations of seedlings were not so strongly substructured as saplings and showed positive autocorrelations at three localities, at two of them (Hrad and Ralsko) in 15 m distance classes and in 5 m distance class at the other (Sopřeč) (Figs. 4, 5, Table 6). The slope of regressions (*b*) between the kinship coefficient (*f_ij_*) and the log of the physical distance between individuals as well as *Sp* statistics corroborate these results (Table 6). When we computed the SGS irrespectively of age classes, we obtained positive autocorrelations at all localities on distances usually up to 25 m (Figs. 4, 5). Similarly, there were positive autocorrelations among individuals of different age classes at three localities (Figs. 4, 5, Table 6), suggesting dispersal distances of *Pinus strobus* seeds at different localities to be around 15 m (Figs. 4, 5).

**Table 6:** Relationships between kinship coefficients and physical distances in *Pinus strobus* populations at which fine scale genetic study was performed.

Locality	Age class	*b*	CI (95%)	*F*_1_	*Sp*
**Bynovec**	Old trees	–0.0004NS	(–0.009–0.008)	0.0115	0.0004
	Mature trees	–0.0064NS	(–0.013–0.011)	0.0818	0.0070
	Saplings	–0.0014NS	(–0.006–0.005)	0.0106	0.0014
	Seedlings	0.0003NS	(–0.012–0.010)	0.0025	–0.0003
	Among classes	–0.0055*	(–0.002–0.002)	0.0188	0.0056
	Overall	–0.0044*	(–0.002–0.002)	0.0177	0.0044
**Hrad**	Old trees	0.0060NS	(–0.013–0.011)	0.0144	–0.0061
	Mature trees	–0.0150*	(–0.008–0.007)	0.0318	0.0155
	Saplings	–0.0104*	(–0.006–0.005)	0.0268	0.0107
	Seedlings	–0.0165*	(–0.012–0.011)	0.0319	0.0170
	Among classes	–0.0078*	(–0.003–0.002)	0.0154	0.0079
	Overall	–0.0086*	(–0.002–0.002)	0.0209	0.0087
**Ralsko**	Old trees	–0.0015NS	(–0.025–0.021)	0.0092	0.0015
	Mature trees	0.0059NS	(–0.013–0.011)	0.0570	–0.0062
	Saplings	–0.0098*	(–0.006–0.005)	0.0133	0.0100
	Seedlings	–0.0061NS	(–0.011–0.009)	0.0033	–0.0033
	Among classes	–0.0064*	(–0.003–0.003)	–0.0012	0.0064
	Overall	–0.0066*	(–0.002–0.002)	0.0024	0.0066
**Sopřeč**	Old trees	–0.0065NS	(–0.010–0.008)	0.0083	0.0066
	Mature trees	0.0030NS	(–0.012–0.010)	–0.0214	–0.0029
	Saplings	–0.0126*	(–0.005–0.004)	0.0197	0.0129
	Seedlings	–0.0076NS	(–0.013–0.011)	–0.0238	0.0075
	Among classes	–0.0034*	(–0.002–0.002)	0.0106	0.0034
	Overall	–0.0047*	(–0.002–0.002)	0.0104	0.0047

Slope of regression (*b*) between the kinship coefficient (*F_ij_*) and the log of the physical distance between individuals, 95% confidence interval (CI) for regression slope, the kinship between pairs of neighbours at the first distance class (*F*_1_) and the *Sp* statistics estimated for different age classes within individual localities of *Pinus strobus*. NS – not significant,

* P<0.05.

## Discussion

This study reports for the first time the patterns of both large-scale (between continent comparison) and fine-scale (spatial genetic structure within populations from outside of the species’ native distribution range) genetic diversity in invasive tree *Pinus strobus*. So far, relevant studies have focused on the species’ native distribution range and have dealt with population genetic structure [100]–[105] or spatial genetic structure [47], [68], [69], [106], [107]. The main findings of the present study are:

1. a comparably high level of genetic diversity present in the native and the adventive ranges, with an absence of strong large-scale genetic structure in both distribution ranges (Table 2). In other words, the amount of genetic diversity detected over a significant part of the native range was also present in the relatively tiny area of the species’ adventive range in the Czech Republic (see Fig. 1).
2. The genetic diversity of trees introduced to the adventive localities approximately one hundred years ago was high enough to ensure the maintenance of high genetic diversity. On the fine scale we found no evidence for any increase in genetic diversity parameters over time; however, we observed the development of fine-scale genetic clustering over time.

### Large-scale Study

#### Genetic diversity in the native vs. the adventive range

The finding of comparably high levels of genetic diversity has two important implications. First, the amount of genetic diversity present in the adventive range is so high that it is difficult to envisage a severe demographic bottleneck having occurred either during or after the introduction to Europe. This finding is consistent with the supply of large amounts of seeds from diverse sources since the beginning of the introduction process (see also below).

Second, even though theory predicts high genetic diversity in the adventive range to be a consequence of multiple introductions, the pattern of genetic diversity in the native range can provide clearer insight into the invasion history. *Pinus strobus* populations in the native range show a pattern of genetic diversity distribution that is less structured than that of many invasive plant species studied so far (but see [108]). Hence, high genetic diversity in the adventive range could be the result of an introduction from one genetically very diverse source or multiple introductions from different sources, or a combination of both. The question arises whether such high genetic diversity could have been generated *in situ* after the introduction of the species in the adventive range. Analysis of fine-scale genetic diversity (see below) within the four localities in the adventive range shows how genetic diversity changed through the invasion process. We did not find any increase over time in the genetic diversity parameters measured, further supporting our explanation that the presence of a high genetic diversity in the adventive range is due to massive introduction of genetically highly diverse material since the beginning of the invasion process. Based on the absence of a difference in genetic diversity between the native and the adventive ranges, together with the lack of structure in the native range, we propose that in this species, genetic structure in the native range did not play a significant role for invasion success in the adventive range as in invasive species having highly genetically structured populations [24], [27].

#### Population structure and identification of sources

Our Bayesian assignment test identified two genetic clusters (Fig. 2). They weakly separated North American native populations from the European, non-native populations (Fig 3). A different picture is presented by the less supported schemes of three or more genetic clusters, in that most individuals were not strongly assigned to any single genetic cluster. Therefore, non-native populations either descended from a cluster that is not represented within the current sample from North America or allele frequencies were strongly shaped during the introduction of genotypes to Europe, resulting in the creation of a novel cluster as a result of invasion. Although we cannot exclude the first scenario despite our sampling design covering a significant part of the native range, our data rather support the latter explanation. Allele frequencies differ between the native and the adventive ranges at all individual loci as well as over all loci, indicating introduction of different individuals in different frequencies.

Interestingly, we did not detect any strong structure even within the native range. There are basically two non-exclusive explanations that can clarify the pattern. (1) *Pinus strobus,* as do some other conifers, shows low population differentiation and considerably high pollen-mediated gene flow [105], [109]–[114]. If a conifer species spreads its pollen effectively over long distances, then low genetic structure would be detected, i.e. pollen-mediated gene flow alone could explain the patterns observed in the native range. (2) The second explanation is linked to the use of *P. strobus* in the timber industry, resulting in disruption of the genetic integrity of original populations due to admixture of populations from different sources. Historically, by the early 1600s, the first colonists were quick to make use of *P. strobus* forest resources in the eastern part of North America, and by the early 1900s the area of destruction of the primeval white pine forests reached from the Atlantic seaboard through the Great Lake states [115], [116]. Some of this cleared land was reforested using seeds and seedlings from different sources, which could have resulted in mixing of genetic material and obscuring the current genetic structure of the species in its native range.

The very low genetic structure of the native populations is worth considering in the light of Taylor and Keller’s study [108] of effects of genetic structure in the native range on the genetic diversity of invasive populations in the adventive range. They found that greater phylogeographic structure in a species’ native range can increase opportunities for admixture among previously isolated lineages in its adventive range, influencing the evolutionary potential there. However, if mixing of individuals from separate native populations occurs prior to introduction, this would weaken the phylogeographic structure, but allow each subpopulation to contain a large portion of the genetic diversity of the whole population. In this scenario, if a sufficient number of genetically diverse individuals are introduced, it obviates the need for multiple introductions. Then, if population regeneration is ensured and inbreeding prevented, as commonly done by foresters, a loss of genetic diversity might not be apparent in the adventive range.

In the case of the introduction of *P. strobus* to the Czech Republic, there is historical evidence of large-scale importations of seeds, which were likely sufficient to largely maintain the genetic diversity of the mixed populations from which they came. For example, there is a historical record that in 1784 10 kg (if the average weight of a *P. strobus* seed is 0.0196 g, than 10 kg is equivalent to introduction of 510,204 seeds) of *P. strobus* seeds were bought and sown in forests in the north-western part of the Czech Republic. These seeds came from Germany with a declared origin in England. Another example is the historical record concerning nearly 32 kg (approximately 1,632,653 seeds) of white pine seeds imported directly from North America in 1800 and planted in the south-eastern part of the Czech Republic [54]. Thus, we know that introductions of new individuals were massive from the very beginning of the cultivation process.

Moreover, we did not see any significant correlation between genetic and geographical distance in either Europe or North America. This pattern is in contrast to studies of other species in which isolation by distance was found in their native ranges and was attributed to a long history of genetic isolation [25]. Our results suggest that present population structure in both the native and the adventive range of *P. strobus* may have been also affected by recent human activities, mainly by the transport of propagules.

#### Evolution within the adventive range

A comparative study between invasive and naturalized populations within the adventive range allows us to assess the consequences of evolutionary events occurring over time. Even if the majority of genetic diversity was introduced from the native to the adventive range, not all introduced populations of *P. strobus* in the Czech Republic can be classified as invasive. Some populations are represented only by old trees and almost completely lack sapling and seedling stages. The test of whether this behavior is influenced by environmental conditions did not reveal any significant relationships. Invasive and naturalized populations only differed in gene diversity (*H*_S_) (see Table 4). Although there is a significant difference, it is so weak that it seems to be biologically irrelevant. However, we obtained a highly significant difference in allele frequencies between invasive and naturalized populations. This may have occurred when different alleles were introduced to different populations, and might be an indication of different seed material used for introduction of *P. strobus* to different areas within the Czech Republic or due to pollen dispersal that distributed alleles across populations within small regions.

### Fine-scale Study

#### Genetic diversity

When a species is introduced to a new range from several genetically distinct populations, further recombination within the adventive range may dramatically increase genetic diversity outside the native range [23]–[25], [27]. However, we are not aware of any published study which has examined changes in genetic diversity in an invasive species outside of its native range over time. Such a study would show whether the initial population at the beginning of an invasion needed admixture to reach its present genetic diversity. Trees are an excellent group for making such comparisons, as different age classes present at a locality represent different stages of the invasion process. What disappears very quickly in other plant life forms (i.e. founding genotypes in the lag-phase of the invasion [29], [33], is still present in trees and can be readily sampled, aged and genotyped. In this study, we took several highly invasive populations of *Pinus strobus* from different parts of the Czech Republic and explored them in detail to answer this question. By comparing the different age classes in four populations, we found that the initial population of old trees did not suffer from low genetic diversity due to the introduction of a low number of individuals. Conversely, our populations of old trees did not differ from younger age classes, with the exception of the locality Hrad, where we detected lower heterozygosity and a consequently higher inbreeding coefficient (*F*_IS_). This means that foresters sowed large amounts of seeds that were genetically diverse enough that no admixture was necessary in this case. Even in highly invasive seedlings, which completely fill the space available in the forest, we did not detect any increase in genetic diversity in comparison with other age classes. These results are concordant with studies performed on a large scale study conclusions presented above.

#### Spatial Genetic Structure (SGS)

Our results concerning SGS corroborate those presented above. Old trees completely lacked any SGS. SGS increased over time and reached its maximum in the sapling stage, but on different distance classes in different populations with the lowest SGS in Bynovec locality (Figs. 4, 5, Table 6). In the seedling stage, however, we detected only a very weak SGS, probably because other factors such as increasing density of surrounding populations, thinning or seed shadow overlap started to gain importance (see below). SGS of *Pinus strobus* populations has been intensively studied in the native range [47], [68], [69], [107]. In these studies, spatial autocorrelation analyses detected weak positive structuring at 10 to 15 m, which fits the isolation by distance model particularly for old growth population. Jones *et al*. [47] attribute weaker patterns observed in *P. strobus* to the longer dispersal distance of seeds and a historical overlap of seed shadows from adults outside of the plot coupled with an overlap of seed shadows from younger, more recently established reproductive adults. They also discuss the possible influence of disturbance and colonization history, mating system and ecological factors on SGS and stress the role of thinning processes, which can weaken the initial structure present in seedlings within the parental population.

Troupin *et al.* [40] analysed the change in SGS of reproductive individuals over a span of 30 years in an expanding *Pinus halepensis* population founded by five reproductive individuals. They found no SGS in the early stages of invasion and suggested the random distribution of genotypes could be the result of density-dependent grazing. In our study on invasive *Pinus strobus,* we also did not detect a SGS in early stages, i.e. in old trees. These trees were planted by foresters, and individual gene combinations were introduced randomly. Old populations therefore cannot have any SGS, and the spatial tree distribution is not an outcome of a biological process but of a human activity. However, many studies of conifer species demonstrate a weak SGS in old-age classes in wind-dispersed species as an outcome of biological processes such as consanguineous mating, low level of pollen flow and short-distance seed dispersal without human influence [44], [47], [69], [117], [118].

Epperson [119] theoretically demonstrated that when the initial distribution of genotypes is random, the degree of spatial autocorrelation quickly increases. In the same way as in the case of *Pinus halepensis* [40], we found a gradual increase in SGS over time that culminated in the sapling stage, in which we detected SGS up to 25 m. Although the data from the native range document SGS at 10–15 m distances [47], [68], [69], [107], the patchiness of invasive populations outside of the native range was wider but not stronger. Many studies demonstrate a strong SGS in the seedling stage compared to the adult stage for other species [49], [120]. This, by contrast, is not the case in invasive populations in the adventive range of *Pinus strobus*. We have observed very weak spatial structuring in the seedling stage at all localities, which should be the result of two processes that are not mutually exclusive, i.e. long-distance seed dispersal and spatial-temporal overlap in seed shadows. Based on these processes, as the population of an invasive tree rapidly increases, propagule pressure increases as well. In combination with the high dispersal capacity of *Pinus strobus* (the species has winged seeds adapted for wind dispersal), seedlings do not form strongly genetically structured populations, as different genotypes are widely mixed due to pollen and seed dispersal over distances longer than 100 m. Hadincová *et al*. [61] and Münzbergová *et al*. [62] showed from different sandstone areas of the Czech Republic that *P. strobus* is able to spread very effectively. They estimated that *P. strobus* can disperse up to 750 m away from the parental source in different localities. As a result, there is great seed shadow overlap from adults within the population as well as adults adjacent to the plot (but which are not included in our analysis). This has the effect of reducing both the level and spatial scale of relatedness in studied populations and is especially pronounced in the seedling stage. On the other hand, 750 m is the maximal detected dispersal distance, a majority of seeds is dispersed up to 100 m apart and that approximately 80% of them are distributed up to 20 m apart. This dispersal pattern may generate fine-scale genetic clustering up to 15 m among age classes.

## Conclusions

In summary, the comparative population genetics of native and adventive *P. strobus* reveals a surprisingly complicated story. The amount of the genetic diversity in the adventive range is attributable to either multiple introductions, similar to findings on other species [23]–[27] or introductions from a single, genetically very diverse, source, or a combination of both. However, as we did not find any population genetic structure in the native range, we suggest that population amalgamation probably first happened in the native range, prior to introduction, due to pollen mediated gene flow and/or human transport of propagules in the 18^th^ century and later. In such a case, there was no need for multiple introductions from previously isolated populations but only several introductions from genetically diverse populations. Moreover, we also found evidence of differentiation between invasive and naturalized populations in the adventive range. Highly invasive populations were clearly distinguished by different allele frequencies. The fine-scale genetic diversity study further supports these results. The genetic diversity of trees introduced to the adventive localities approximately one hundred years ago was high enough to ensure the maintenance of high genetic diversity. At the fine scale within the invasive populations, we found no evidence for any increase in genetic diversity parameters over time. Furthermore, in invasive populations, we observed the development of fine-scale genetic clustering over time. This occurred at the maximum distance of 25 m, at which old trees completely lacked any spatial genetic structure that increased over time and reached its maximum in the sapling stage. These results support the hypothesis that rather than admixture, a single introduction from a genetically diverse source or multiple introductions from different sources of similar genetic diversity pattern, or a combination of such events, was likely responsible for the high genetic diversity of *P. strobus* populations in the adventive range.
